# Effect of Playing Position, Match Half, and Match Day on the Trunk Inclination, G-Forces, and Locomotor Efficiency Experienced by Elite Soccer Players in Match Play

**DOI:** 10.3390/s20205814

**Published:** 2020-10-14

**Authors:** José M. Oliva-Lozano, Elisa F. Maraver, Víctor Fortes, José M. Muyor

**Affiliations:** 1 Health Research Centre, University of Almería, 04120 Almería, Spain; josemuyor@ual.es; 2 Faculty of Computer Science, Multimedia and Telecommunications, Universitat Oberta de Catalunya, 08018 Barcelona, Spain; elisafm@uoc.edu; 3 Unión Deportiva Almería, 04007 Almería, Spain; vifosa@gmail.com; 4 Laboratory of Kinesiology, Biomechanics and Ergonomics (KIBIOMER Lab.), Research Central Services, University of Almería, 04120 Almería, Spain

**Keywords:** football, posture, match analysis, load, team sports, inertial measurement units

## Abstract

The rapid growth of wearable sensors has allowed the analysis of trunk kinematics during the match, which is necessary for having a better understanding of the postural demands of soccer players. However, some contextual variables may have an impact on the physical demands of the players. This study aimed to analyze the effect of three contextual variables (playing position, match half, and match day) on the sagittal trunk inclination, G-forces, and locomotor efficiency experienced by soccer players in match play. Then, wearable sensors were used to collect the trunk kinematics during 13 matches. Firstly, positional differences were found on the trunk inclination (*p* = 0.01) and the G-forces experienced by the players (*p* < 0.001). For example, the greatest and lowest trunk inclination was found for FW (~34.01°) and FB (~28.85°) while the greatest and lowest G-forces were found for WMF (1.16 G) and CD (1.12 G), respectively. However, there were no positional differences in the locomotor efficiency (*p* = 0.10). Secondly, the match half had a significant effect on the trunk inclination (*p* = 0.01) and the G-forces experienced by the players (*p* < 0.001) with significantly lower values observed during the second half. No differences between halves were found on the locomotor efficiency for any playing position (*p* = 0.41). Finally, no significant effect of match day on any variable was observed. This investigation is one of the first steps towards enhancing the understanding of trunk kinematics from elite soccer players. The positional differences found on the trunk inclination and G-forces imply that the development of position-specific training drills considering the postural demands is necessary to prepare the players not only for the physical demands but also for successful performance in the field of regard. The resistance to fatigue needs to be trained given the differences between halves.

## 1. Introduction

In recent years, a number of investigations on the physical demands of elite soccer players have been conducted [1,2,3,4,5] since the governing body of soccer, which is FIFA, allowed the use of electronic performance and tracking systems in official matches [6]. These investigations frequently analyze parameters such as total distance, high-speed running distance, a total of accelerations or decelerations performed by the players during the match because these parameters help coaches to adapt the training load and the training drills by having a better understanding of match demands [1,2,3,4,5]. However, elite soccer matches are characterized by fast paces of play which require high demands of perceptual-motor skills of the players (e.g., continuous interaction with teammates in a specific space and time to dribble the ball while considering the opposition players) [7,8,9]. In consequence, recent investigations on other team sports (e.g., field hockey) have suggested that a better understanding of the postural demands of the players is necessary [10,11].

One of the variables that give an insight into the postural demands is the sagittal trunk inclination of the player while running [10,11]. This variable is highly associated with the low back pain [12], hamstring injury [13], hip and knee energetics in running [14], and patellofemoral joint stress [15]. For example, running at a controlled speed (e.g., 3.4 m/s) with an excessive upright posture has been associated with greater patellofemoral joint stress compared to running with forward trunk flexion [15]. In addition, the trunk inclination is not only related to the development of musculoskeletal injuries but it also may explain the performance in the field of regard [10,16]. Then, the trunk inclination may be considered as an important variable since soccer players usually play the ball with the feet and their ability to perform in the field of regard may be affected as well [10].

Furthermore, the analysis of the trunk accelerations through the G-forces experienced by the players may provide meaningful information about the players’ performance [17,18]. Soccer is a team sport characterized by high-intensity actions (e.g., intermittent sprints, changes of direction, body impacts, jumps, and landings), which implies that measuring the trunk accelerations is necessary [1,2,19]. Since inertial sensors allow the quantification of the trunk accelerations in the three axes of movement, recent investigations have suggested the use of G-forces as an external workload indicator [18]. The trunk plays an important role as “shock absorber”, but the magnitude (i.e., G-forces) and amount of shocks on the musculoskeletal system may increase the risk of injury [20,21].

Thus, the integration of inertial sensors (e.g., accelerometers) with global positioning systems (GPS) has become a trending method for workload monitoring in team sports [18]. Many studies support the practical implementation of accelerometry monitoring through variables such as the locomotor efficiency ratio (i.e., player load/total distance covered) [18,22]. This is a very practical variable from a performance standpoint since a player that accumulates more load than another player may be indicative of a reduced locomotor efficiency if both covered the same distance [22]. Moreover, the locomotor efficiency ratio has been identified as a representative variable of fatigue and injury risk in match play [22].

In consequence, the analysis of the postural demands through the trunk inclination, the G-forces experienced by the players and the locomotor efficiency ratio is necessary. In addition, contextual variables such as playing position, match half and match day, which usually have an impact on the physical demands of elite soccer players [2,22,23,24], need to be considered. However, limited data have been published to date. To the best of the authors’ knowledge, no studies are available to date regarding the analysis of trunk inclination and the G-forces experienced by the players in competitive match-play. Thus, this study aimed to analyze the effect of playing position, match half, and match day on the sagittal trunk inclination, G-forces, and locomotor efficiency ratio of elite soccer players in match play.

## 2. Materials and Methods

### 2.1. Study Design

This study used a cohort design for 13 microcycles in which one match per microcycle (i.e., weekly periods counting from the day after the match to the following match) was played by an elite soccer team from LaLiga 123. These microcycles belonged to the last phase of the season. Wearable sensors, which contained inertial measurement units and GPS technology, were used to collect the trunk kinematics in match play. The data collection was authorized by the club and informed consent for volunteer participation in the study was given. The approval from the institutional bioethics committee was obtained.

### 2.2. Participants

Fifteen elite male soccer players (27.1 ± 3.9 years old; 75.6 ± 5.6 kg; 1.8 ± 0.1 m) took part in this study. They belonged to specific playing positions: central defenders (CD, 26 match observations), full-backs (FB, 19 match observations), wide-midfielders (WMF, 18 match observations), midfielders (MF, 17 match observations), and forwards (FW, 14 match observations). However, the players who could not participate in the total duration of the match were not included in the study to avoid the effect of pacing strategies [2,3,25,26]. In addition, goalkeepers were excluded from the analysis given the differences in the activity-profile [27].

### 2.3. Procedures

**Figure 1:**
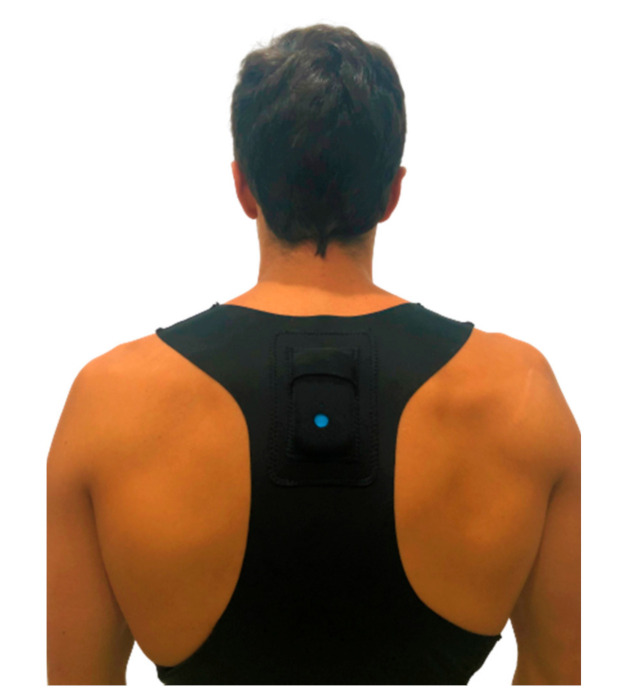
Tracking system placed in the back pocket of a chest vest.

WIMU Pro units (RealTrack Systems, Almería, Spain) were used as electronic performance tracking systems to collect the data during the matches. These wearable units, which were placed in the back pocket of a chest vest on a vertical position (Figure 1), contain inertial sensors (four 3D accelerometers, three 3D gyroscopes, one 3D magnetometer) recording data at 100 Hz and GPS technology recording data at 10 Hz. Each unit was calibrated before the start of the match following the manufacturer’s instructions [24]. The data collected by the units were transferred to SPro (RealTrack Systems, Almería, Span) at the end of the matches to download the “Intervals Pro” report in addition to the raw data from “ATTITUDE EULER Z” and “ACELT” channels.

**Figure 2:**
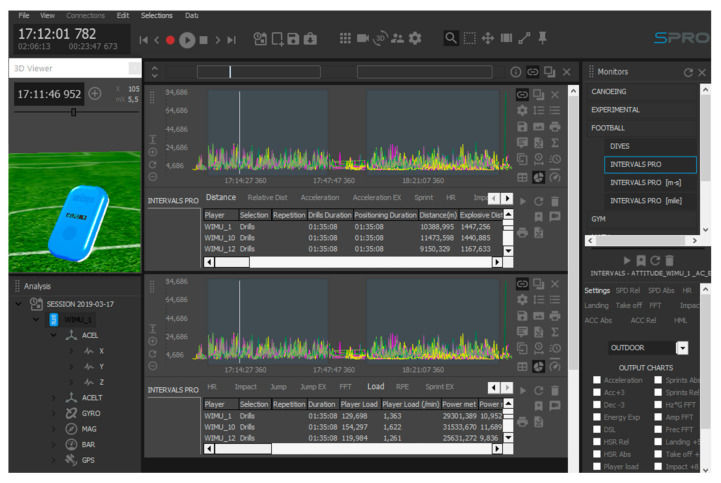
Data collected by the tracking system on the Intervals Pro report (RealTrack Systems, Almería, Spain), which were used to calculate the locomotor efficiency ratio.

The process to obtain these data was as follows: first, open the session file; second, create the first and second half drills using the “Select mode” on SPro; third, launch “ATTITUDE EULER Z” channel, “ACELT” channel, and “Intervals Pro” to the central panel; forth, click on “Export” to download the data. The “Intervals Pro” report was used to obtain the total distance covered (in meters) and the player load, which is an accelerometer-derived variable that derives from movements registered in the *x*-, *y*-, and *z*-axis [28]. These variables allowed the calculation of the locomotor efficiency (i.e., locomotor efficiency = player load/total distance covered in meters) [29] (Figure 2).

**Figure 3:**
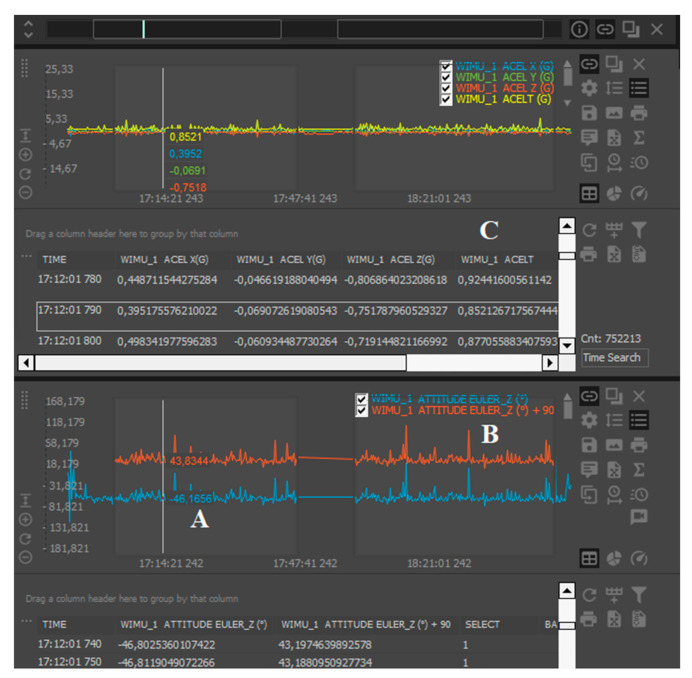
Raw data from “ATTITUDE EULER Z” (**A**), “ATTITUDE EULER Z plus 90” to obtain the right position (**B**), and “ACELT” (**C**).

The “ATTITUDE EULER Z” was used to analyze the sagittal trunk inclination (in degrees) [30] (Figure 3A). Since the upright posture equals 0° and the original data collected by the unit reported 90° for the same position, the researchers applied a simple formula, which was the data value from “ATTITUDE EULER Z” plus 90, to obtain the right position (Figure 3B). Thirdly, the “ACELT” data, which reported the resultant vector of the G-forces from horizontal and vertical movements registered by the three axes (*x*, *y*, *z*) of the accelerometers (ACELT formula = $\sqrt{x^{2}+y^{2}+z^{2}}$ ) [31] (Figure 3C).

### 2.4. Statistical Analysis

First of all, the descriptive statistics for the trunk inclination, G-forces and locomotor efficiency ratio were calculated by playing position, match half, and match day. Then, the equality of variances was calculated through Levene’s test and the sphericity was calculated through Mauchly’s test (*p* < 0.05 in all variables). A linear model with a mixed-design analysis of variance for repeated measures was run. Trunk inclination, G-forces and locomotor efficiency ratio were set as dependent variables. Then, the Bonferroni post hoc was used to compare the results from the dependent variables between playing positions, match halves, and match days. The confidence interval (CI) and Cohen’s *d* were reported for the pairwise comparisons. In addition, the partial eta-squared (*ηp*^2^) was calculated to express the amount of variance accounted for the independent variables. The statistical analysis was performed on SPSS Statistics (IBM Corp., Armonk, NY, USA) with the level of significance set at *p* ≤ 0.05.

## 3. Results

### 3.1. Sagittal Trunk Inclination

**Figure 4:**
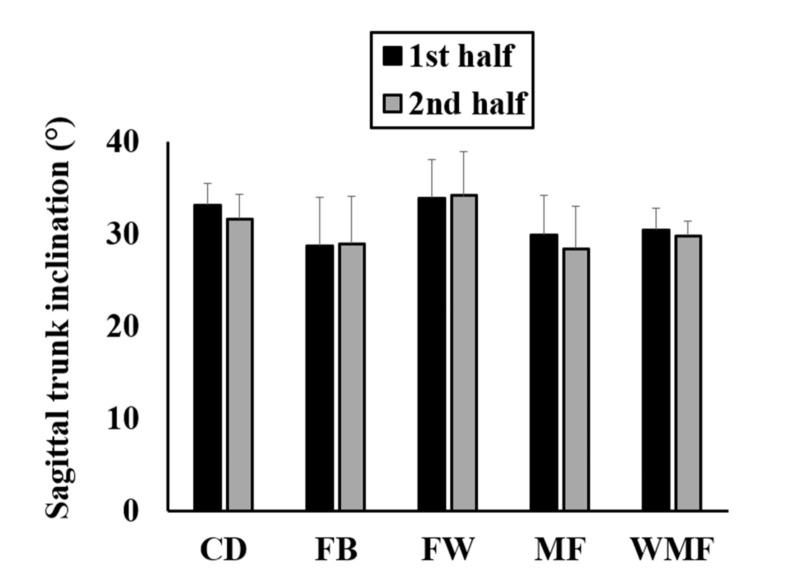
Sagittal trunk inclination of elite soccer players based on playing position and match half.

Figure 4 shows the mean sagittal trunk inclination and standard deviation by playing position and match half. The playing position had a significant effect on the sagittal trunk inclination (*F*_(4, 32)_ = 4.44; *p* = 0.01; *ηp*^2^ = 0.36). Regarding the match half, it had a significant effect on the trunk inclination (*F*_(1, 32)_ = 9.05; *p* = 0.01; *ηp*^2^ = 0.22). Then, the interaction between match half and playing position was significant too (*F*_(4, 32)_ = 3.80; *p* = 0.01; *ηp*^2^ = 0.32). Specifically, the sagittal trunk inclination during the first half was significantly greater compared to the second half in CD (~1.52°; *p* = 0.01; CI = 0.75–2.28; *d* = 0.60) and MF (~1.45°; *p* = 0.01; CI = 0.45–2.45; *d* = 0.33).

In addition, FW showed significantly greater sagittal trunk inclination than FB in the first half (~5.09°; *p* = 0.04; CI = 0.13–10.05; *d* = 1.06). During the second half, FW also showed significantly greater inclination than FB (~5.49°; *p* = 0.02; CI = 0.74–10.25; *d* = 1.05), MF (~6.43°; *p* = 0.01; CI = 1.55–11.31; *d* = 1.22), and WMF (~4.99°; *p* = 0.04; CI = 0.18–9.80; *d* = 1.21). However, there was no significant effect of match day on the sagittal trunk inclination (*F*_(12, 32)_ = 0.81; *p* = 0.64; *ηp*^2^ = 0.23) so no significant differences in the trunk inclination (*p* > 0.05) were observed between match days for any playing position.

### 3.2. G-Forces

**Figure 5:**
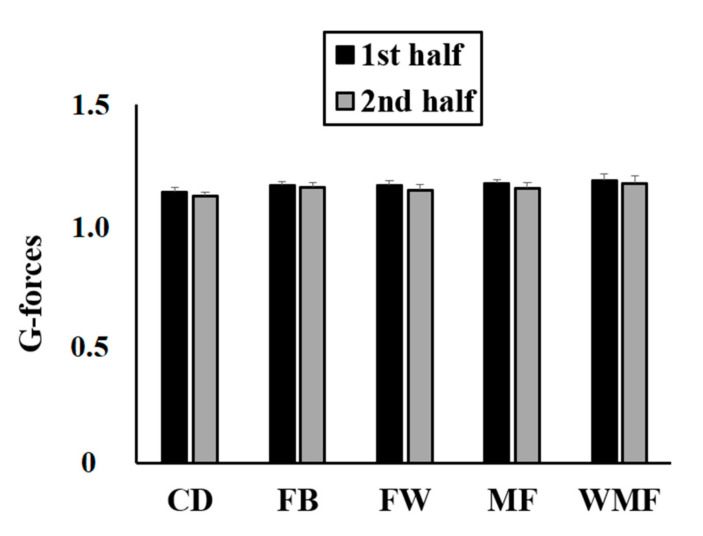
G-forces experienced by elite soccer players based on playing position and match half.

The G-forces experienced by the players in match play (Figure 5) were significantly influenced by the match half (*F*_(1, 32)_ = 182.58; *p* < 0.001; *ηp*^2^ = 0.85) and the playing position (*F*_(4, 32)_ = 14.56; *p* < 0.001; *ηp*^2^ = 0.65). The interaction between match half and playing position was also significant (*F*_(4, 32)_ = 5.15; *p* = 0.003; *ηp*^2^ = 0.39). In this regard, the G-forces were lower during the second half of the matches compared to the first half for CD (~0.02 G; *p* = 0.01; CI = 0.013–0.021; *d* = 0.87), FB (~0.01 G; *p* = 0.01; CI = 0.002–0.012; *d* = 0.40), FW (~0.02 G; *p* = 0.01; CI = 0.015–0.026; *d* = 0.93), MF (~0.02 G; *p* = 0.01; CI = 0.013–0.024; *d* = 1.01), and WMF (~0.01 G; *p* = 0.01; CI = 0.007–0.018; *d* = 0.41).

During the first half of the matches, CD reported significantly lower G-forces than FB (~0.03 G; *p* = 0.03; CI = 0.002–0.049; *d* = 1.43), MF (~0.04 G; *p* = 0.001; CI = 0.014–0.063; *d* = 2.05), WMF (~0.06 G; *p* = 0.001; CI = 0.029–0.078; *d* = 2.03), and FW (~0.03 G; *p* = 0.03; CI = 0.002–0.054; *d* = 1.47). In addition, WMF showed significantly greater G-forces than FB (~0.03 G; *p* = 0.03; CI = 0.002–0.055; *d* = 0.97) in the first half. During the second half of the matches, CD reported significantly lower G-forces than FB (~0.04 G; *p* = 0.001; CI = 0.013–0.058; *d* = 1.91), MF (~0.04 G; *p* = 0.001; CI = 0.013–0.058; *d* = 1.57), and WMF (~0.06 G; *p* = 0.001; CI = 0.035–0.080; *d* = 2.28). The WMF showed significantly greater G-forces than FW (~0.03 G; *p* = 0.01; CI = 0.01–0.06; *d* = 1.12) during the second half. However, the G-forces experienced by the players did not significantly differ between match days (*F*_(12, 32)_ = 1.41; *p* = 0.21; *ηp*^2^ = 0.35).

### 3.3. Locomotor Efficiency Ratio

**Figure 6:**
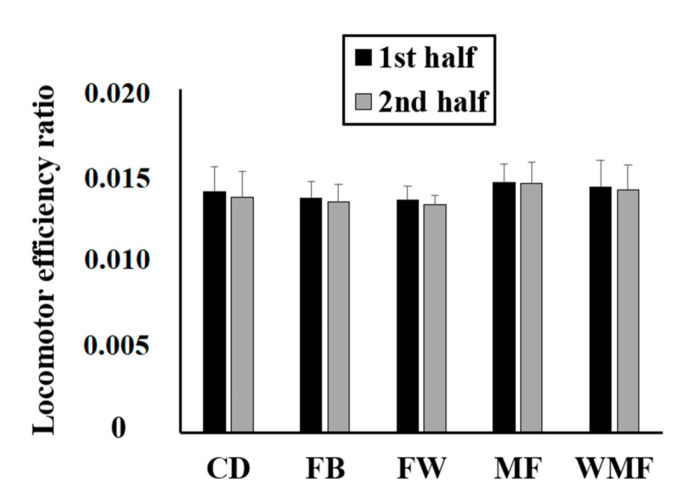
Locomotor efficiency ratio from elite soccer players based on playing position and match half.

Figure 6 shows the locomotor efficiency ratio by playing position and match half. Although the match half (*F*_(1, 32)_ = 9.67; *p* = 0.004; *ηp*^2^ = 0.23) had a significant effect on the locomotor efficiency ratio, the effect of playing position (*F*_(4, 32)_ = 2.12; *p* = 0.10; *ηp*^2^ = 0.21) and the interaction between match half and playing position was not significant (*F*_(4, 32)_ = 1.03; *p* = 0.41; *ηp*^2^ = 0.11). No differences (*p* > 0.05) were found between playing positions. In addition, match day had no significant effect on the locomotor efficiency ratio experienced by the soccer players (*F*_(12, 32)_ = 0.47; *p* = 0.92; *ηp*^2^ = 0.15).

## 4. Discussion

The main purpose of this investigation was to analyze the effect of playing position, match half, and match day on trunk kinematics of elite soccer players in match play. This investigation, which is one of the first steps towards enhancing the understanding of trunk kinematics from elite soccer players, presents several key findings. Firstly, positional differences were found on the sagittal trunk inclination and the G-forces experienced by the players. However, there were no positional differences in the locomotor efficiency ratio. Secondly, the sagittal trunk inclination and the G-forces experienced by the players were significantly greater during the first half of the matches, while no differences were found for the locomotor efficiency ratio. Finally, no significant differences between matches were observed on the sagittal trunk inclination, G-forces, and locomotor efficiency ratio.

When it comes to the positional differences observed for the trunk inclination and the G-forces experienced by elite soccer players, it is important to mention that soccer is a team sport in which the positional role has a significant influence on the physical demands of the players [3,5,32,33]. The results showed trunk inclination values lower (i.e., more upright postures) than other team sports such as field hockey, in which the mean trunk inclination in match play was ~45° and little differences between the playing positions were observed (defenders: ~43.5°; midfielders: ~44.2°; strikers: ~46.4°) [10]. The trunk flexion position lets the players carry out soccer-specific movements (e.g., running, passing, or controlling the ball) but these actions may move the field of regard down and limit the visual exploration [10,16]. In addition, soccer is a team sport with continuous running actions, accelerations, decelerations, and changes of directions for each position [2,19], which induce constant trunk accelerations. Then, the differences in the activity-profile from each position may explain why there were positional differences in G-forces in match play. In consequence, the development of position-specific training drills considering the trunk inclination and G-forces experienced by the players is necessary to prepare not only for the physical demands (e.g., training trunk flexion postures with constant G-forces, which may avoid low back pain) [12] but also for successful performance in the field of regard [10,16]. However, this consideration does not seem to be so important for the locomotor efficiency ratio since no positional differences were found, which confirms the results reported by a previous investigation [22].

Regarding the effect of match half on the trunk kinematics variables, this study showed that elite soccer players reported greater sagittal trunk inclination and G-forces during the first half of the matches while no differences between halves were found for the locomotor efficiency ratio. Previous studies, which have analyzed the influence of match half on the activity-profile of elite soccer players, observed that the physical performance decreased during the second half of the matches [24,25,26,34]. For example, the performance in high-intensity actions, which increase trunk inclination angle, is reduced during the second half of the matches [24,34]. This may be one of the reasons why our CD, MF, and WMF adopted significantly more upright postures during the second half. In this regard, the declines in physical performance have been linked to a depletion of muscle glycogen towards the end of the match [26,35]. These observations have significant practical implications for strength and conditioning coaches since the resistance to fatigue needs to be trained through trunk strength exercises, trunk mobility (e.g., spine sparing exercises) in addition to lumbopelvic control exercises [12,36]. However, match half did not significantly affect the locomotor efficiency ratio of the players and these results are in line with the only investigation on this ratio available to date [22]. This study found that specific phases of the match led to different locomotor efficiency ratios but it did not report differences between halves [22]. For instance, the ratio was greater during the last 15 min of the first half compared to the first 15 min of the second half, but this ratio was greater during the last 30 min of the match compared to first 15 min. In this regard, a previous study also concluded that the skill-related performance measures did not significantly change between halves [26]. Therefore, future investigations are necessary to examine the relationship between locomotor efficiency ratio and the physiological response of elite soccer players.

In addition, this study found that the match day did not affect the sagittal trunk inclination, G-forces, or locomotor efficiency ratio since there were no significant differences between matches. These results imply that these variables may be closely related to the player’s biomechanics and other contextual variables (e.g., match day, opponent team) may have not played a significant role in the sample investigated. For example, the match-to-match variability on total distance covered (~5%) is usually lower than the variability on high-speed running distance (~53%) [37]. Unfortunately, these research questions regarding the effects of match day on trunk kinematics have limited answers to date since no study has previously analyzed the influence of match day on the sagittal trunk inclination or G-forces experienced by elite soccer players, and only one study on the locomotor efficiency ratio is available [22]. In fact, contrary to what was previously shown in our results, a significant variability (~21%) on the locomotor efficiency between matches was observed [22]. The reason for these contradictory results is not clear, but it is worth mentioning that there was an important difference on the total of matches analyzed since this study included 86 matches [22] while ours was limited to 13 league matches.

Unfortunately, limited data is available to date regarding the trunk kinematics of soccer players in the course of a match. The analysis of the trunk inclination, G-forces experienced by the players and locomotor efficiency when running or sprinting should be indispensable for hamstring injury prevention [13]. The reason is that excessive trunk and pelvic motion in addition to the lack of lumbopelvic control during the swing phase of the running action is closely related to the injury risk [13]. Most studies usually analyze the number of horizontal trunk accelerations per intensity zones based on the activity-demands profile of the sport (e.g., soccer: 0–5 G, 5–6 G, >9 G; basketball: 0–3 G, 3–5 G, 5–8 G, >8 G; rugby: 0–6 G, 6–7 G, 7–8 G, 8–10 G, 10–12 G, >12 G; endurance trail running: 1G ranges from 0 to 30 G) [38,39,40,41]. However, these studies neither analyzed the total of trunk accelerations nor the average magnitude of these trunk accelerations (i.e., triaxial G-forces), which has been considered as a key external load indicator in professional soccer by a principal component analysis [2].

Therefore, the lack of investigations on the trunk kinematics of soccer player limited the discussion of our results. Nevertheless, this study has several limitations, which need to be considered. For example, the sample included in this study was specific to one men’s professional soccer team during 13 league matches. In addition, not all the team players could be included because only those who played the total match were considered for the analysis. Other playing positions such goalkeepers were not analyzed. Goalkeepers were excluded from the analysis given the differences in the activity-profile. In consequence, further investigations are needed to be done considering these limitations in addition to other physiological variables (e.g., heart rate), which may help to understand the role of fatigue on the sagittal trunk inclination, G-forces and locomotor efficiency experienced by the players.

Furthermore, it is to mention that the data were collected by inertial sensors and GPS technology instead of optical tracking systems. Although the tracking systems used in this study have been considered as valid, reliable and suitable instruments to measure position-related variables [42,43] and trunk inclination in the sagittal plane [30], the gold standard technology for measuring position and orientation of human body segments are optical tracking systems [30,44,45]. However, the use of optical tracking systems is limited to laboratory settings for technical reasons [30,44,45] and thus, tracking systems with inertial sensors and GPS technology have vast implications for human activity monitoring. In consequence, future investigations analyzing the trunk kinematics and postural demands should consider the advantages of using these wearable sensors as well as their validity and reliability for measuring each parameter. For example, these wearables should include methods that compensate the drift error (e.g., multi-sensor fusion from accelerometers, gyroscopes, and magnetometers) to provide more accurate data [46,47,48,49]. In this regard, an investigation suggested the use of several inertial sensors, which were placed on both shanks, thighs, sacrum, and sternum, as a successful method for reducing the drift error in highly dynamic movements [45]. Nevertheless, this last method does not seem to be useful from a practical perspective in professional soccer since wearing different sensors around the body may be uncomfortable and dangerous for the players [50].

## 5. Conclusions

The data collected by inertial measurement units and global positioning systems may be used for having a better understanding of the postural demands of professional soccer players. This may be considered as the first study analyzing the trunk kinematics of professional soccer players in official matches. In addition, a novel approach was conducted by analyzing the effect that different contextual variables (e.g., playing position, match half, and match day) had on the postural demands. Although the only investigations that have analyzed the trunk kinematics have been carried out in field hockey players, our study with soccer players tried to improve the applied methodology since these investigations presented two major limitations: the sample of matches (6 matches and 1 match, respectively) [10,11] and the use of different instruments for data collection within the same investigation [10].

The positional differences found on the sagittal trunk inclination and the G-forces experienced by the players imply that the development of position-specific training drills considering the postural demands is necessary to prepare the players not only for the physical demands (e.g., training trunk flexion postures with constant G-forces) but also for successful performance in the field of regard. Strength and conditioning coaches must include trunk strength exercises with special focus on flexion positions as well as other compensatory exercises for trunk extension muscles, which balance trunk flexors and trunk extensors, before and after match days. Specifically, it would be important to add perturbations to stimulate trunk accelerations (~1–2 G) during trunk strength exercises. In addition, the resistance to fatigue needs to be trained given the differences in the postural demands observed between halves. Finally, it is recommended that the soccer players exercise trunk mobility (e.g., spine sparing exercises) before and after the match with a special focus on the posterior chain.
